# A common protein export pathway in malaria parasites

**DOI:** 10.1186/1475-2875-9-S2-I3

**Published:** 2010-10-20

**Authors:** Brendan Crabb, Hayley Bullen, Sarah Charnaud, Silvia Haase, Justin Boddey, Alan Cowman, Tania de Koning-Ward, Paul Gilson

**Affiliations:** 1 Burnet Institute, Melbourne 3004, Australia; 2 School of Medicine, Deakin University Waurn Ponds campus, Geelong 3217, Australia; 3 Walter & Eliza Hall Institute, Parkville 3052, Australia

## 

Protozoan parasites that cause malaria export hundreds of proteins into their host red blood cell cytosol, and some even beyond that to the extracellular environment. These proteins have a wide range of functions that are crucial to parasite virulence and/or parasite survival in the human host. It has been thought for some time that a common link to all these proteins is the mechanism by which they are exported. Recently, we have revealed much of how this export occurs, including the discovery of a novel translocon through which exported proteins must pass. As a common portal for many essential proteins this translocon becomes a strongly validated drug target.

